# The Channel-Activating Protease CAP1/Prss8 Is Required for Placental Labyrinth Maturation

**DOI:** 10.1371/journal.pone.0055796

**Published:** 2013-02-06

**Authors:** Edith Hummler, Aline Dousse, Audrey Rieder, Jean-Christophe Stehle, Isabelle Rubera, Maria-Chiara Osterheld, Friedrich Beermann, Simona Frateschi, Roch-Philippe Charles

**Affiliations:** 1 Department of Pharmacology and Toxicology, University of Lausanne, Lausanne, Switzerland; 2 University Institute of Pathology, University of Lausanne, Lausanne, Switzerland; 3 ISREC (Swiss Institute for Experimental Cancer Research), School of Life Sciences, EPFL (Ecole Polytechnique Fédérale de Lausanne), Lausanne, Switzerland; University Hospital Hamburg-Eppendorf, Germany

## Abstract

The serine protease CAP1/Prss8 is crucial for skin barrier function, lung alveolar fluid clearance and has been unveiled as diagnostic marker for specific cancer types. Here, we show that a constitutive knockout of CAP1/Prss8 leads to embryonic lethality. These embryos presented no specific defects, but it is during this period, and in particular at E13.5, that wildtype placentas show an increased expression of CAP1/Prss8, thus suggesting a placental defect in the knockout situation. The placentas of knockout embryos exhibited significantly reduced vascular development and incomplete cellular maturation. In contrary, epiblast-specific deletion of CAP1/Prss8 allowed development until birth. These CAP1/Prss8-deficient newborns presented abnormal epidermis, and died soon after birth due to impaired skin function. We thus conclude that a late placental insufficiency might be the primary cause of embryonic lethality in CAP1/Prss8 knockouts. This study highlights a novel and crucial role for CAP1/Prss8 in placental development and function.

## Introduction

Proteases or peptidases are enzymes able to catalyze the hydrolysis of peptide bonds representing about 2% of all proteins in human [1] or mouse [2]. Proteases are often involved in signaling cascades, where a zymogen is cleaved and converted into its active form, allowing amplification but also fine-tune regulation of a signal. Moreover, endogenous protease inhibitors are present within a cell, giving a high level of complexity for these proteins *in vivo* [3]. They are involved in many processes, from the classical digestive function, to cellular signaling and specific protein cleavage. Caspases are important in apoptosis, matrix metallo-proteinases (MMPs) are involved in tissue remodeling, thrombin plays a key role during blood clotting, while deubiquitinating enzymes (DUBs) are required for sub-cellular localization of proteins. Serine proteases are implicated in cell growth, survival, invasion, angiogenesis and inflammation and appear to be involved in human disease, including cancer. Therefore, there is a growing interest in developing therapies to control the activity and expression of proteases [4], e.g., inhibitors of the angiotensin-converting enzyme, in the treatment of hypertension.

Mouse channel activating protease 1, also termed CAP1/Prss8 for protease serine S1 family member 8, has been named for its ability to increase ENaC-mediated sodium current *in vitro* [5], [6] and *in vivo* [7], [8]. According to the peptidase classification [2], it belongs to the subfamily A of the S1 chymotrypsin family as trypsin and chymotrypsin. The S1 family is characterized by a catalytic triad, histidin, aspartate and serine (HDS), that are gathered to form the catalytic site by folding of the protein [9]. CAP1/Prss8 belongs to a subgroup of S1 serine proteases that are anchored to plasma membranes via a glycosyl-phophatidylinositol linkage (GPI-anchored) located at the lipid rafts that are important for cell-cell communication [10]–[12]. Human CAP1/Prss8 has been previously isolated from seminal fluids of prostate cancer patients and named prostasin [13]. First described as a cancer marker, it has later been linked to reduced tumor growth as seen by reduced invasiveness of breast [14], gastric [15] and prostate cancer [16], [17].

Recent studies suggest that CAP1/Prss8 is linked to matriptase (Prss14; MT-SP1) in a protease cascade [18]–[21]. Activation of CAP1 by the protease hepsin [22] has also been described. Equally associated with cancer [23], matriptase seems to act as a tumor suppressor gene in vitro [24], an effect proposed to be mediated through CAP1/Prss8 [25]. On the other hand, potential inhibitors of CAP1/Prss8 are the hepatocyte growth factor activator inhibitor-1 (HAI-1) [26], and nexin-1 (PN-1), which inhibits CAP1/Prss8-induced sodium current in *Xenopus* oocytes [27].

Using mice carrying a conditional knockout allele of CAP1/Prss8 [28], we have previously demonstrated that CAP1/Prss8 is required for normal skin barrier function resulting in postnatal death due to severe dehydration when CAP1/Prss8 is deleted in skin [29]. In addition, increased expression of CAP1/Prss8 induces severe skin defects [30]. Moreover, CAP1/Prss8 is involved in water and salt balance in lung alveolar type II cells [7]. Furthermore, we have recently described two spontaneous animal models carrying mutations in the CAP1/Prss8 gene, presenting skin defects and constitutively reduced basal activity of ENaC [8].

In the present study, we unveil a novel and important role of CAP1/Prss8 during embryonic development. Our data clearly illustrate that proper expression of CAP1/Prss8 in the placenta triggers late placental labyrinth maturation, thereby maintaining late gestation embryonic development and allowing the embryo to develop to term.

## Results

### A Constitutive Knockout of CAP1/Prss8 Results in Embryonic Lethality

**Figure 1:**
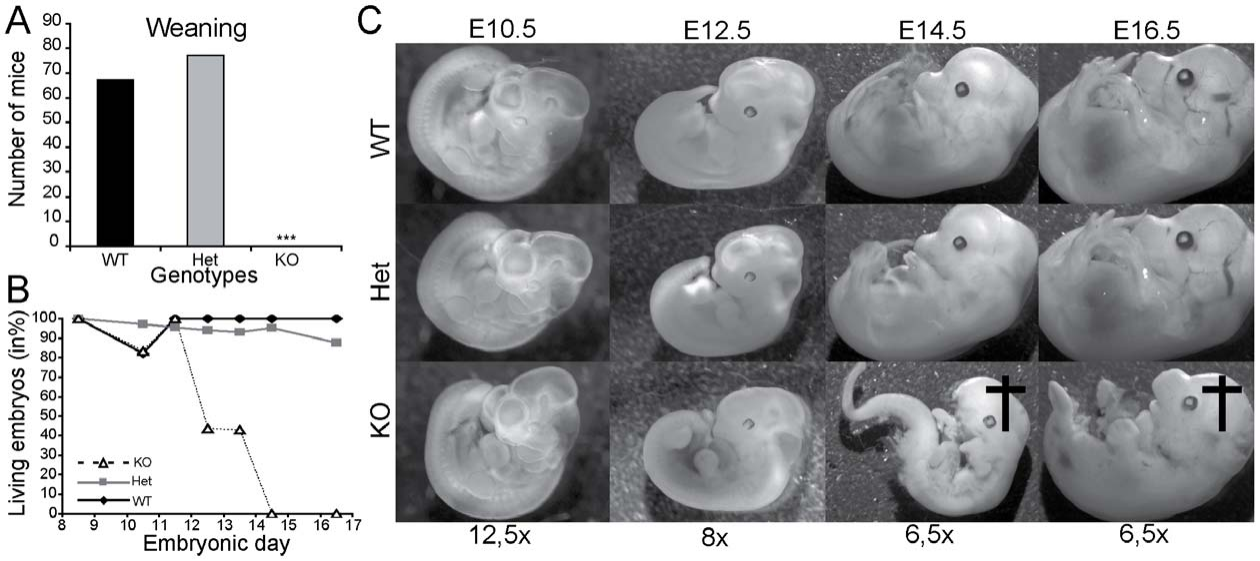
Constitutive CAP1/Prss8 inactivation results in embryonic lethality. Distribution of genotypes following heterozygote breeding. Number of wildtype (WT, black) CAP1/Prss8 heterozygous mutant (Het, grey) and knockout (KO, white) mice at weaning (**A**). Survival curves in percentage of wildtype (black diamonds), heterozygous mutant (grey spares) and knockout (black diamonds) littermate embryos between E8.5 and E16.5. Note, that no knockout embryo survived beyond E14.5 (**B**). (**C**) Representative pictures of gross morphology of embryos at different developmental stages from wildtype (WT), heterozygous mutant (Het) and homozygous mutant (KO) mice. The black cross indicates dead/partially degraded embryos; ***P<0.001.

Serine proteases are implicated in many physiological and pathological processes. In order to uncover new roles of the membrane-bound serine protease CAP1/Prss8, we generated constitutive knockouts of CAP1/Prss8 (Δ/Δ) by intercrossing heterozygous mutant mice (Δ/+). CAP1/Prss8 knockout mice were missing at weaning (**Fig. 1A**), and at birth (data not shown), suggesting embryonic lethality. To identify the cause we dissected embryos from E8.5 to E16.5 and determined the representation of each genotype (**Fig.1B**). CAP1/Prss8 knockout mice were found underrepresented, and none of the knockout embryos was found alive from E14.5 onwards as determined by lack of heart beating and visible pulsatile blood vessels. Examination of the knockout embryos did not reveal any obvious developmental defects, like neural tube closure, or heart malformations (**Fig. 1C**). Most often, only the placenta and/or the degraded dead CAP1/Prss8 κνοχκουτ embryo were found (**Fig. 1C**).

### Late Differentiation Defect is Detected in CAP1/Prss8 Knockout Placentas

**Figure 2:**
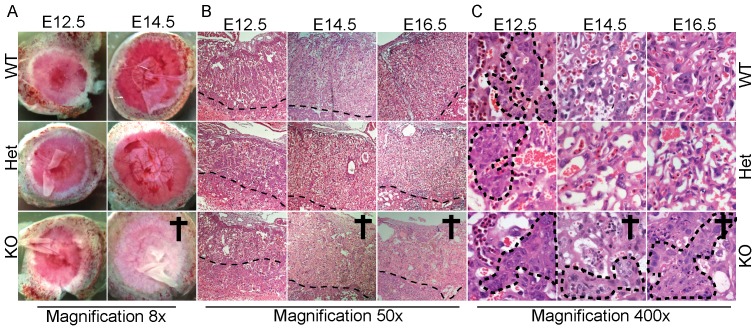
CAP1/Prss8 knockout placentas present a late differentiation defect. Representative pictures of placenta. (**A**) Placental discs at E12.5 and E14.5. H&E stainings of sagittal sections of placentas at E12.5 and E14.5 from wildtype (WT), heterozygous (Het) and knockout (KO) embryos at (**B**) low (x50) and at (**C**) high magnification (x400). From day E12.5 onwards, maturation from cytotrophoblasts to syncytiotrophoblasts is observed in wildtype and heterozygous mutant but not in knockout. The dashed line indicates the limit between the labyrinth and the decidua in **B**, and surrounds the cytotrophoblasts in **C**.

While wildtypes and heterozygotes showed an organized arborization of the vessels starting from the umbilical cord, placentas of knockout embryos exhibited only a couple of large vessels and marginal ones (**Fig. 2A**). Histopathological analysis of E12.5 to E14.5 embryos revealed that knockout placentas are less organized with less marked placental cotyledons, and fewer blood vessels at E14.5. At E12.5 and E14.5, the labyrinth size did not differ amongst the experimental and control groups (E12.5; wt: 875±120 pixel; het: 890±267 pixel; ko: 732±149 pixel versus E14.5; wt: 945±259 pixel; het: 1346±65 and ko: 887±275 pixel) (see also **Fig. 2B** **).** At the cellular level, a regular transition of the placenta histology can be observed in wildtypes. At E12.5, the placenta is mainly composed of cuboidal cytotrophoblasts that are fusing to form a syncytium from E10.5 to E16.5. This results in a thinner separation between maternal blood (small anucleate erythrocytes) and the fetal blood (large nucleated erythrocytes). In addition, the surface of exchange progressively increases. This process is observed in heterozygotes as well. In contrast, knockout placentas showed a majority of cuboidal cytotrophoblasts at E12.5 and this was maintained at all later time points, indicating incapability to form a proper syncytium (**Fig. 2C**).

All together, these data highly suggest an altered development of the placenta of knockout embryos from E12.5 onwards.

### Increased Transient CAP1/Prss8 Expression is Required for Normal Placenta Maturation

**Figure 3:**
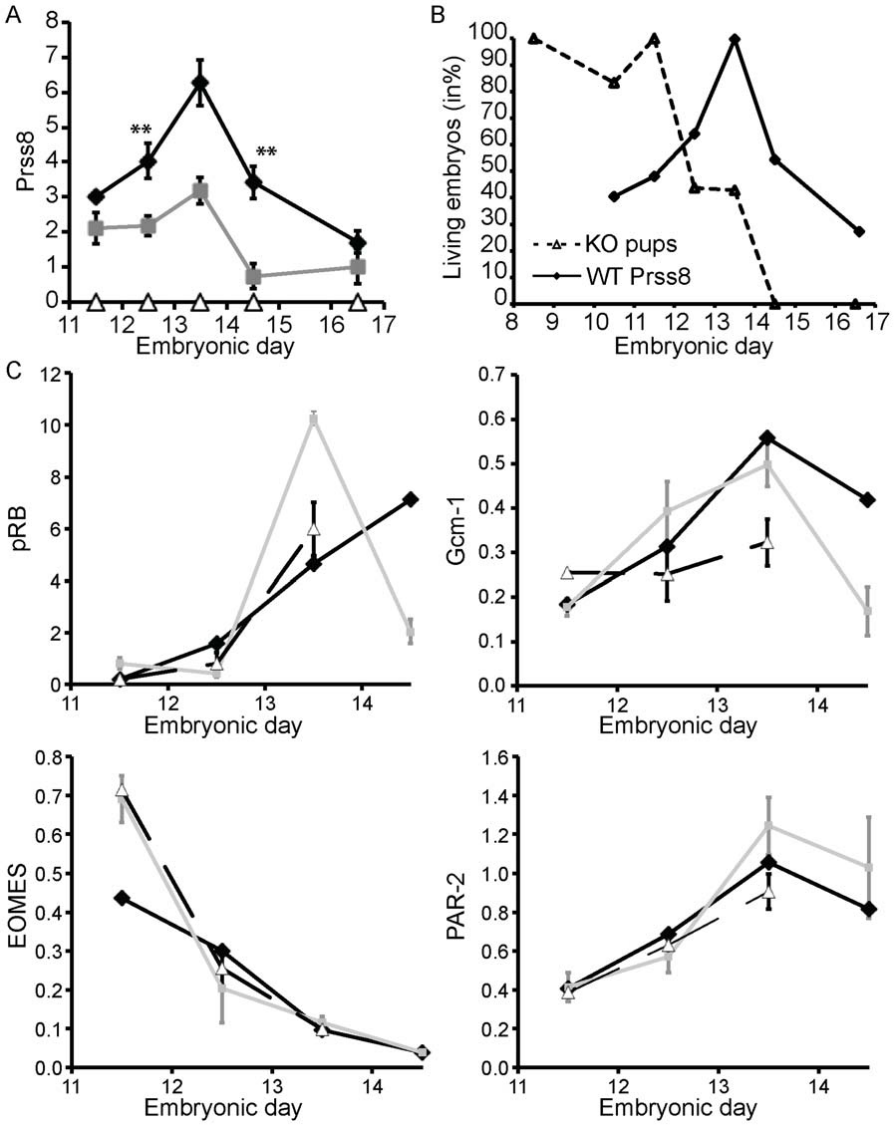
mRNA transcription profile of placental-specific markers. Placental expression of CAP1/Prss8 (Prss8) (**A**) of wildtype (WT, black diamond), heterozygous (Het, grey box), and knockout (KO, open triangle) embryos. **Shows the statistical difference between wildtype and heterozygous level. (**B**) Overlay of the CAP1/Prss8 peak of expression in wildtype placenta (solid line) and the survival curve of knockout embryos (dotted line). (**C**) Relative mRNA expression levels of *Par2, pRb, Eomes and Gcm1* were determined in placenta from wildtype, heterozygous mutant and knockout embyros from E11.5 to E14.5, each time point representing the average value measured from at least 3 animals.

To evaluate the importance of CAP1/Prss8 in the placenta, we followed its expression level by quantitative RT-PCR. In wildtype, CAP1/Prss8 mRNA transcript expression in the placenta increases from E11.5 onwards, reaching a peak around E13.5 before declining to basal levels (**Fig. 3A**). No CAP1/Prss8 expression was detected in placenta from knockout embryos, while heterozygotes showed an intermediate expression level (**Fig. 3A**). In the embryo itself, the expression level of Prss8 was increasing 2-fold between E12.5 and 14.5 but overall remained 10- to 100-fold lower than in the placenta. At time points considered in this study, no transient peak of Prss8 expression was detected in the knockout embryo (data not shown; see also **Fig. 3A**). Interestingly, the peak of CAP1/Prss8 expression in wildtype placentas overlapped with the reduced survival and the abnormal placental development in knockouts (**Fig. 3B**).

To study further the consequences of CAP1/Prss8-deficiency, we analyzed the mRNA transcript expression of the markers pRb, Gcm1, EOMES and PAR2. pRb, EOMES and Gcm1 are transcription factors involved in placental differentiation and/or development. Absence of retinoblastoma protein (pRb) leads to a placental defect and embryonic death at E14.5 [29]. In our study, pRb expression in wildtype placentas increased from E11.5 to E14.5, and this was not affected by the absence of CAP1/Prss8 (**Fig. 3C**). The T-box transcription factor Eomesodermin (Eomes) is involved in the formation of the extra-embryonic trophoblast lineage [30]. Eomes is necessary for gastrulation and early events of placenta formation. We found that Eomes expression in the placenta decreased with time showing no difference between wildtype and knockout (**Fig. 3C**). The Protease-Activated Receptor-2, PAR2, is a G-coupled receptor found to be activated by CAP1/Prss8 in mouse embryo and in the skin [30]. Our results show that PAR2 is equally expressed in the placenta of both wildtype and knockout embryos (**Fig. 3C**). The knockout of the glial cell missing 1 (Gcm1) transcription factor leads to a failure of branchomorphogenesis and labyrinth formation from E8.5 onwards. Interestingly, Gcm1 promotes the fusion of the cytotrophoblast and syncytiotrophoblast [31]. In wildtype placenta, Gcm1 mRNA expression increased till E13.5, and did not significantly differ from the knockout placenta (**Fig. 3C**).

### Specific Inactivation of CAP1/Prss8 in the Epiblast is Compatible with Embryonic Development Until Birth

**Figure 4:**
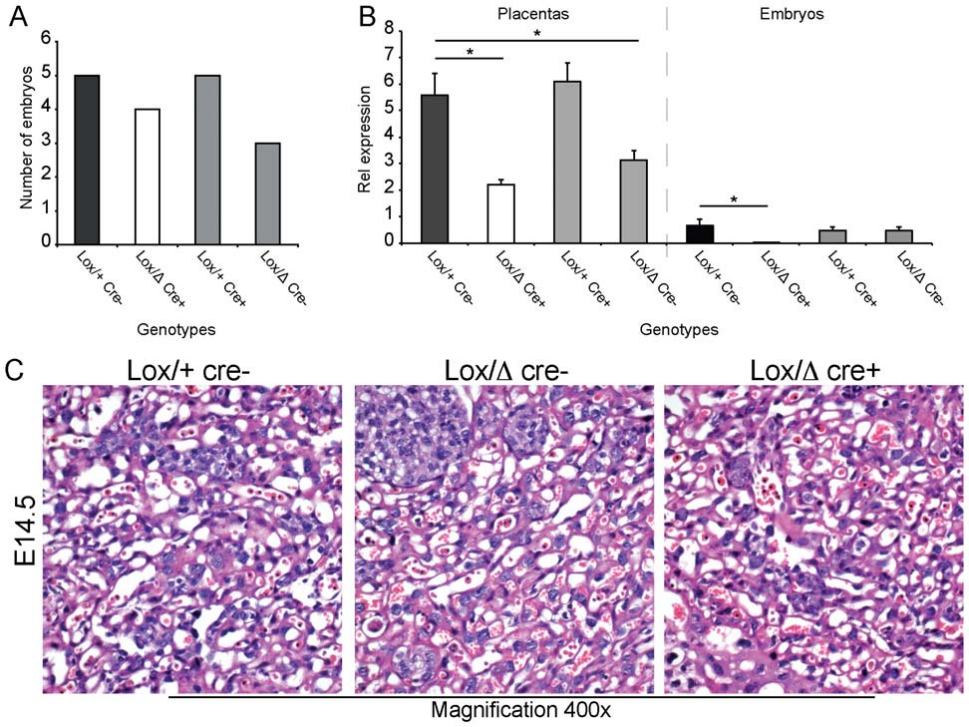
CAP1/Prss8 knockout embryos present normal placentas. Epiblast-specific CAP1/Prss8 knockout (Lox/Δ cre+) pups were obtained by mating *CAP1/Prss8* ^Δ*/+*^ *;Sox2::Cre^Tg/+^* to *CAP1/Prss8^lox/lox^* mice. (**A**) Genotype distribution of embryos recovered at E14.5 and relative quantification of CAP1/Prss8 mRNA expression levels in placentas and embryos (**B**). *CAP1/Prss8^lox/^* ^Δ^ *;Sox2::Cre^Tg/+^*, embryo-specific knockout (Lox/Δ Cre+), white bar; *CAP1/Prss8^lox/^* ^Δ^ (Lox/Δ Cre-) and *CAP1/Prss8^lox/+^;Sox2::Cre^Tg/+^*, heterozygous mutant (Lox/+ Cre+), grey bar; *CAP1/Prss8^lox/+^* wildtype (Lox/+ Cre-); black bar. Note that the CAP1/Prss8 mRNA expression in the knockout (Lox/Δ Cre+) embryos is abolished while the corresponding control (Lox/+ Cre+) placentas show heterozygous expression levels. (**C**) H&E staining of sagittal placenta sections at E14.5 from controls (*CAP1/Prss8^lox/+^*; Lox/+ cre-), heterozygotes (*CAP1/Prss8^lox/^* ^Δ^; Lox/Δ) and embryo-specific knockouts (*CAP1/Prss8^ lox/^* ^Δ^ *;Sox2::Cre^Tg/+^*; Lox/Δ cre+).

To distinguish whether the observed embryonic lethality is primarily caused by embryonic death or placental failure, we targeted CAP1/Prss8 deletion specifically in the epiblast using Sox2::Cre transgenic mice, aiming to delete CAP1/Prss8 in the embryo proper, but not in the placenta. Expression from the Sox2::Cre transgene induces recombination in the epiblast from E6.5 onwards but not in extra-embryonic cell types. Following CAP1/Prss8^Δ/+^; Sox2::Cre^Tg/+^ x CAP1/Prss8^lox/lox^ breeding, we genotyped embryos and found both knockout (CAP1/Prss8^lox/Δ^;Sox2::Cre^Tg/+^), and control animals at E14.5 (CAP1/Prss8^lox/Δ^, CAP1/Prss8^lox/+^ and CAP1/Prss8^lox/+^;Sox2::Cre^Tg/+^, **Fig. 4A**). CAP1/Prss8 mRNA transcript expression was detected by quantitative RT-PCR in the placenta of the conditional knockouts while it was completely absent in the embryo itself (**Fig. 4B**). The placentas of the heterozygous mutant (CAP1/Prss8^lox/+^; Sox2::Cre^Tg/+^) embryos retained intermediate levels of CAP1/Prss8 expression compared to knockout (CAP1/Prss8^lox/+^ and CAP1/Prss8^lox/Δ^;Sox2::Cre^Tg/+^), as expected from the genotype (**Fig. 4B**). Histologically, the placentas of all genotypes presented a comparable and normal development at E14.5 with syncytiotrophoblasts composing the majority of a matured labyrinth (**Fig. 4C**). Moreover, the CAP1/Prss8^lox/Δ^; Sox2::Cre+ knockout embryos were morphologically indistinguishable from the controls (data not shown).

**Figure 5:**
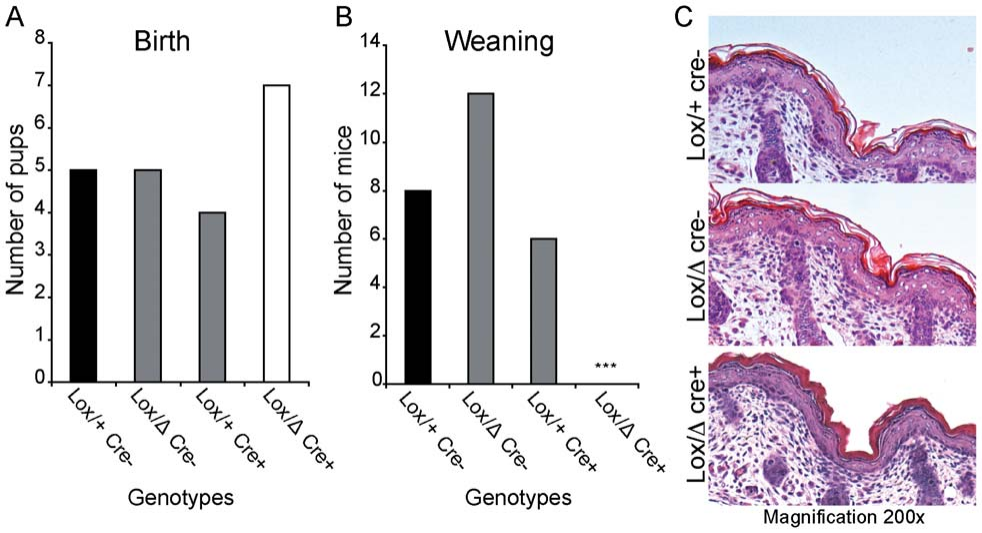
CAP1/Prss8 knockout embryos are born and exhibit an abnormal epidermis. Genotype distribution of pups at birth and at weaning (*CAP1/Prss8* ^Δ*/+*^ *;Sox2::Cre^Tg/+^* x *CAP1/Prss8^lox/lox^*). Two litters were taken right after birth (n = 21 pups, **A**) or following weaning (3 week-old; n = 26 pups, **B**) and genotyped. (**C**) H&E staining of sagittal backskin sections of one-day-old pups from controls (*CAP1/Prss8^lox/+^*; Lox/+ cre-), heterozygous (*CAP1/Prss8^lox/^* ^Δ^; Lox/Δ cre-) and embryo-specific knockouts (*CAP1/Prss8^lox/^* ^Δ^ *;Sox2::Cre^Tg/+^*; Lox/Δ cre+). The latter displayed a compact stratum corneum (hyperkeratosis) indicative of a skin barrier defect. Four additional litters were genotyped at weaning; magnification x200.

To assess the consequences of the lack of CAP1/Prss8 in the embryos during the perinatal period, two litters were monitored until birth. Conditional knockouts were born according to the expected Mendelian distribution (**Fig. 5A** **)**. Histological observation of the skin of the CAP1/Prss8^lox/Δ^;Sox2::Cre^Tg/+^ pups right after birth showed hyperkeratosis (**Fig. 5C**). This is a characteristic phenotype of skin-specific CAP1/Prss8 knockout animals as has been previously described and leads to early postnatal death due to severe dehydration [29]. Four more litters from the same breeding (CAP1/Prss8^Δ/+^; Sox2::Cre^Tg/+^ x CAP1/Prss8^lox/lox^) were kept until weaning, and no knockout (CAP1/Prss8^lox/Δ^; Sox2:: Cre^Tg/+^) mice could be detected at that time point (**Fig. 5B**).

We concluded that the lack of CAP1/Prss8 in the embryo is compatible with normal placenta function and embryo development demonstrating that the embryonic death of constitutive CAP1/Prss8 knockout animals is primarily caused by placenta failure.

## Discussion

Placenta and embryo are highly dependent on each other and form a tightly regulated system with a placenta progressively adapting to the increasing requirements of the embryo. To determine the consequences of CAP1/Prss8 deficiency during embryonic development, we analyzed in detail a complete CAP1/Prss8 knockout mouse model, and found that the absence of the protein leads to embryonic lethality. The present study shows a novel important role of the membrane-bound serine protease CAP1/Prss8 in placental development during late gestation.

Looking at different time points during embryonic development, we found that 50% of the knockout embryos were dead at day E12.5 or E13.5, and 100% from day E14.5 onwards (**Fig. 1**). We therefore concluded that CAP1/Prss8 deletion resulted in increased embryonic lethality. No obvious developmental delay and/or defect was observed in E12.5 embryos. Cell proliferation (Ki67) and TUNEL staining, and vascularization of the placenta, as visualized by the staining for the endothelial cell marker CD31, was not different from the wildtype (data not shown), thus rather suggesting a functional defect. From that time point onwards, we found the knockout embryos at varying developmental stages, and they often presented signs of advanced post-mortem degradation as illustrated in **Fig. 1C**, and thus were not informative for the cause of lethality (**Fig.1**).

The placenta from CAP1/Prss8 knockout embryos appears pale and lacks the arborescence of vessels as observed in wildtype embryos at E14.5 (**Fig. 2**). From day E12.5 to E16.5, wildtype placentas undergo major modifications. The labyrinth of the placenta allows the maternal and embryonic gas and nutrient exchange [31]. Trophoblastic cells develop from cuboidal and proliferative cytotrophoblasts to thin and highly differentiated syncytiotrophoblast cells, thereby increasing the exchange surface and capacity (**Fig. 2**). The placenta from CAP1/Prss8 knockouts apparently failed to undergo this maturation step, and histological observation indicated that cytotrophoblasts are still mainly present and are not replaced by syncytiotrophoblasts, preventing the development of blood vessels. The development of knockout placentas appeared to be modified from E12.5 onwards. We cannot exclude, however, that functional impairment may precede histopathological changes in the knockout placentas.

To evaluate the importance of CAP1/Prss8 expression in the placenta, we measured mRNA levels by quantitative RT-PCR. We first noticed that placental CAP1/Prss8 expression in wildtypes peaks at E13.5, at a time point where only less than 50% of knockout embryos survived (**Fig. 3**). It is worth noting that the maternal placenta shows only neglectable expression of CAP1/Prss8 (data not shown). The peak of CAP1/Prss8 expression (at E13.5) parallels the Gcm1 expression peak in wildtype. Gcm1 is a transcription factor that promotes the late phase of differentiation of the trophoblast lineage [32] by regulating the activity of the placental-specific enhancer of the aromatase gene (CYP19) [33]. Interestingly, it was not significantly different between the experimental and control group (**Fig. 3C**), and might thus not represent the primary cause of lethality. The expression level of Eomes that is involved in the development of the trophoblast lineage did not differ in the knockout and control placenta (**Fig. 3C**). Moreover, the mRNA transcript expression of the protease-activated receptor PAR2, recently identified as downstream target of CAP1/Prss8 [30], was not altered at that developmental stage. Hepatocyte growth factor activator inhibitor (HAI-1) can form inhibitor complexes with several trypsin-like serine proteases and is required for mouse placental development and embryo survival [34]. HAI-1 is coexpressed with matriptase and CAP1/Prss8 in chorionic trophoblasts, and ablation of HAI-1 led to a complete loss of undifferentiated chorionic trophoblasts after E9.5 [34]. In contrary, genetic ablation of matriptase activity in HAI-1-deficient embryos restored the integrity of chorionic trophoblasts and development to term [34]. Till E12.5, we did not detect any alterations in trophoblast development in our CAP1/Prss8-deficient embryos, suggesting that their functions may overlap, but they do not seem to be part of the same serine protease cascade.

A known target of CAP1/Prss8 is the epithelial sodium channel ENaC. Whereas a recent study suggested a role of ENaC in decidual cells for embryo implantation, mice lacking ENaC activity seem not affected during embryonic development and are born according to the expected Mendelian distribution [35], [36]. Thus, we consider a major implication of ENaC in placental development as rather unlikely.

The close relationship between the embryo and placenta still leaves the question of the primary cause, leading to the death of both the placenta and the embryo. We here hypothesize that the placenta fails to develop which results in embryonic death, but on the other hand, when the embryo dies, the placental development may stop likewise. To test this hypothesis, we deleted CAP1/Prss8 exclusively in the epiblast, while placental CAP1/Prss8 expression was maintained. At birth, all expected genotypes were present following the Mendelian distribution (**Fig. 5A**), while no constitutive knockout developed to term (**Fig. 5**). mRNA analyses showed that we indeed successfully abolished CAP1/Prss8 expression in the embryo when it was maintained in placenta (**Fig. 4B**). The results presented in the paper thus clearly show that CAP1 is required for prenatal development, although not within the embryo itself. Pups lacking CAP1 only in the embryo (but not in the placenta) were even born according to a Mendelian distribution (**Fig. 5**). This clearly shows that the lack of CAP1/Prss8 during embryonic development is without any consequence for the embryo. Nevertheless, those pups were born with an hyperkeratosis as seen in newborns lacking CAP1/Prss8 expression in the epidermis [29] (**Fig. 5**), thus causing death shortly after birth. None of these pups reached the weaning age (**Fig. 5**), and the phenotype thus recapitulates the features of an epidermal-specific CAP/Prss8 knockout.

In conclusion, our study shows that constitutive knockout of CAP1/Prss8 leads to embryonic lethality due to placental failure. In addition, CAP1/Prss8 expression within the embryo seems not required for its development. CAP1/Prss8 is detected in primate [37] and in human placenta in villi at the level of the trophoblasts and placental cell lines [38]. Our data strongly suggest that balanced placental expression of human CAP1/PRSS8 might be relevant in the context of placental maturation.

## Materials and Methods

### Animals

Experimental procedures and animal maintenance followed federal guidelines and were approved by local cantonal authorities. CAP1/Prss8 constitutive knockouts were obtained by the breeding of heterozygous males and females (CAP1/Prss8^Δ/+^). These mice and their genotyping have been described previously [28], [29]. Sox2::Cre mice were kindly provided by Elizabeth Robertson (University of Oxford, UK). This Sox2::Cre^Tg/+^ transgene is inducing recombination in the epiblast [39], [40]. Male CAP1/Prss8^Δ/+^ Sox2::Cre^tg/+^ mice were crossed with CAP1/Prss8^lox/lox^ females in order to obtain embryo-specific knockouts. Cre genotyping was performed using PCR (primer1, sense, 5′-CCTGGAAAATGCTTCTGTCCG-3′, primer 2, antisense, 5′-CAGGGTGTTATAAGCAATCCC-3′). All experiments were performed coded.

### Ethics Statement

All animal work was conducted according to Swiss national guidelines. All mice were kept in the animal facility under UNIL animal care regulations. They were housed in individual cages at 23±1°C with a 12-h light/dark cycle. All animals were supplied with food and water ad libitum. This study has been reviewed and approved (authorization no. 1793.1 to EH) by the “Service de la consommation et des affaires vétérinaires” of the canton of Vaud, Switzerland.

### Extraction of Embryonic and Perinatal Tissues

Breedings were started in the evenings, and females were checked for vaginal plugs the next morning, with the morning of plug defined as E0.5. Embryos were recovered at indicated time points, and embryos and placentas were dissected individually. Visceral yolk sacs of each embryo were washed in PBS and processed for genomic DNA extraction and genotyping. Placenta and embryos were separated, rinsed in PBS, and either fixed in formalin over night prior to paraffin embedding, or snap-frozen in liquid nitrogen prior to protein/RNA extractions. Embryos were counted as alive when a beating heart was observed (from E10.5 onwards). Additionally, from E12.5 onwards, viability of embryos was assessed by color and visible pulsative vascularization.

### Histology

2–3 µm sections were obtained from paraffin blocks and stained with H&E. Labyrinth thickness was measured using ImageJ.

### Quantitative RT-PCR

Embryonic and placental total RNAs were extracted with Qiazol (Qiagen, Basel, Switzerland). The reverse transcription reaction was performed with 1 µg of RNA mixed in a final volume of 20 µl with oligo dT(20) primer (5 µM), dNTP (500 µM), buffer (50 mM TrisHCl pH 8.3, 75 mM KCl, 3 mM MgCl2; 5 mM DTT), and 40 U of SuperscriptII (Invitrogen, Basel, Switzerland). The reaction was incubated at 42°C for 1 h and finally stopped by denaturing the enzyme for 10 min at 70°C. The cDNA was diluted five times before measurements. Quantitative real-time PCRs were performed by TaqMan PCR using the Applied Biosystems 7500 (primers and probes used detailed in table 1). The universal TaqMan mix (2x) and SybR green mix (2x) were purchased and used according to the manufacturer’s instructions (Applied Biosystems, Foster City, CA). The quantifications were calculated by measuring the ΔΔCt normalized to ß-actin level. All measurements were done in duplicate. All values are expressed in relative expression (in % of the actin expression level). Primers were used as listed in **Table 1**.

**Table 1:** Primers used for quantitative PCR.

TaqMan	Prss8	For	CCCATCTGCCTCCCTGC
		Rev	CCATCCCGTGACAGTACAGTGA
		Probe	CCAATGCCTCCTTTCCCAACGGC
	Gcm-1	For	TACAGCTCGGACGACAGGAA
		Rev	AGCAGCTGGGCCATGGGCAA
		Probe	CCCCAGGCATGACTTCTTGA
	ß-Actin	For	AGGTCATCACTATTGGCAACGA
		Rev	CACTTCATGATGGAATTGAATGTAGTT
		Probe	TGCCACAGGATTCCATACCCAAGAAGG
SybR green	pRB	For	CTGGCCTGTGCTCTTGAAGT
		Rev	TGAGGCTGCTTGTGTCTCTG
	Eomes	For	ACATAAAAATTCAGAATCTCTAAAAAG
		Rev	GATTCATATCTTGGAGATATTCTGT
	PAR2	For	ACCTGGCAAGAAGGCTAAGA
		Rev	GACTGAAGCTCTACCAGGGC
	ß-Actin	For	TGGAATCCTGTGGCATCCATGAAA
		Rev	TAAAACGCAGCTCAGTAACAGTCCG

### Calculation and Statistics

All data are expressed as means ± SEM. Individual groups were compared by using the *t* test for unpaired comparisons. The Mendelian distributions were analysed by chi-square tests. A level of *P*<0.05 was accepted as statistically significant for all comparisons. *P<0.05, **P<0.01, ***P<0.001.
